# Older Men's Participation in Cognitive Training: A Review.

**DOI:** 10.1093/geroni/igab046.2822

**Published:** 2021-12-17

**Authors:** Estela González, Carmen Requena, Paula Alvarez

**Affiliations:** Universidad de León, León, Castilla y Leon, Spain

## Abstract

Background: Cognitive training for healthy older adults living in a community is an essential resource that allows them to live at home for as long as possible. Objective: The purpose of the review is to examine the degree of participation of males and females in longitudinal studies of cognitive training. Moreover, we want to identify if these studies include the gender variable in their analyzes or reflect on its importance. Method: This review considered longitudinal cognitive training studies were published in English and Spanish and conducted with healthy older adults living in a community. Results: The Advanced Cognitive Training for Independent and Vital Elderly (ACTIVE) study (in English) and the Memoria Mejor (MM) Longitudinal Study (in Spanish), both illustrate the trend of sex/gender treatment of the studies reviewed: a) high participation of older people seventy and more years b) recruiting stratified by age and sex; c) males are disproportionately underrepresented in cognitive training studies [24% - 14%]; d) the evaluation measures (baseline, follow-up, and final) and dropout data are provided but not stratified by age and sex/gender. Conclusions: Researchers demonstrate awareness about the impact of sex/gender differences but do not focus on it. Understanding sex/gender differences are necessary for understanding not only that these differences occur, but also why they occur; this will allow policies or intervention programs with approaches that are more equitable for both sexes/genders to be formulated.

